# A rare case report of solitary neurofibroma arising in the male breast

**DOI:** 10.1097/RC9.0000000000000293

**Published:** 2026-02-13

**Authors:** Erik L. Parkhurst, Samson M. Chan

**Affiliations:** a School of Medicine, International Program, Universidad Autónoma de Guadalajara, Zapopan, Jalisco, Mexico; b Department of Surgery, Insight Hospital and Medical Center, Chicago, United States of America

**Keywords:** benign peripheral nerve sheath tumor, case report, male breast lesion, neurofibroma

## Abstract

**Introduction and Importance:**

Primary tumors of the chest wall are fairly uncommon and typically involve the mammary glands. Additional breast lesions usually involve the soft tissues, with neurofibromas comprising a significantly low amount of cases that skew toward female patients. A neurofibroma of the male breast is an exceedingly rare occurrence; however, most cases are associated with Neurofibromatosis Type 1 (NF1). This report aims to present the case of a solitary neurofibroma in the breast of a male patient in the absence of a diagnosis of Neurofibromatosis, a situation with only three prior cases documented in the literature. In addition, we review the literature and management approach for this rare occurrence.

**Presentation of Case:**

A 41-year-old male with a PMHx of medication-controlled HTN and migraines noticed a small firm mass in his left breast years ago that recently started causing discomfort with deep palpation despite no reported significant changes in size or development of additional symptoms. Preliminary diagnostic studies (diagnostic mammogram, targeted ultrasound, ultrasound-guided biopsy) illustrated a circumscribed, spiculated oval mass 3 cm from the nipple–areolar complex at the 9-o’clock position, with a biopsy report indicative of a neurofibroma. The patient underwent elective surgical excision of the lesion, and the surgical pathology report additionally confirmed the diagnosis of a neurofibroma.

**Clinical Discussion:**

Neurofibromas are benign peripheral nerve sheath tumors that originate from the endoneurium, the connective tissue of nerve sheaths. Solitary neurofibroma of the male breast in the absence of a diagnosis of neurofibromatosis is a substantially rare occurrence, with only three prior confirmed cases documented. Current literature suggests surgical excision as the mainstay of management, regardless of their low risk of malignant transformation.

**Conclusion:**

To our knowledge, this is only the fourth case of its kind and the only one to provide gross imagery and two separate confirmatory pathology reports. Implications of the findings include continued preferred management via surgical excision, remaining awareness of rare tumors, and additional evidence for the possible prevalence of rare male breast tumors that may have been previously not considered.

## Introduction

A solitary neurofibroma of the male breast in the absence of a diagnosis of Neurofibromatosis is an exceedingly rare occurrence, with only three prior cases documented in the literature^[^1–3^]^. This report covers a recently documented case in a 41-year-old male confirmed through ultrasound-guided biopsy and subsequent surgical pathology report.

Excluding gynecomastia and carcinoma, pathological lesions of the male breast are rare and are infrequently encountered in clinical practice. Mesenchymal neoplasms, across all subtypes, account for approximately 0.5%–1% of all breast tumors in the general population^[^4^]^. Neurofibromas represent an even smaller percentage of this incidence rate, and with the added context of a male patient in the absence of neurofibromatosis, the implied rarity of this case cannot be understated.

**HIGHLIGHTS**

- Solitary neurofibroma of the male breast in the absence of neurofibromatosis is an exceptionally rare entity.
- This is only the fourth case of its kind and the only one to provide gross imagery with two separate instances of confirmatory pathology reporting.
- Lesion developed as a singular homogenous mass without involvement of the nipple–areolar complex and was devoid of notable deformity of the breast architecture and overlying skin.
- Current literature suggests surgical excision as the mainstay of management, regardless of their low risk of malignant transformation.
- Rarity of the location has also led to a recommendation of genetic counseling for all mammary neurofibroma patients.

This work has been reported in line with the SCARE criteria^[^5^]^.

## Case report

A 41-year-old male with a PMHx of medication-controlled HTN and migraine headaches presented to the outpatient surgical clinic for evaluation of a left breast lump that was first noticed approximately 4 years prior. Patient reported that he first appreciated the lump while attempting to lift objects at work and continued to monitor its size in the subsequent years. No notable change in size was reported since the initial onset, but he did admit to recently increasing pain on occasion in the affected area with forceful palpation, which prompted discussions with his primary care provider. He denied any discomfort during sedentary activities or during daily activities. Lump noted to be soft and movable, and there were no reported visible changes to the nipple, nipple discharge, surrounding skin changes, or additional symptoms. Patient denied any family history of male breast lesions or breast cancer in any family members. Prior to evaluation for possible surgical excision, he was sent by his primary care provider for a male Bilateral Digital Diagnostic Mammogram and Targeted Left Breast Ultrasound about one month before surgical consultation. After suspicious findings were reported on the initial imaging report, he was sent 3 weeks later to receive an Ultrasound-guided Biopsy of the Left Breast with Marking Device Insertion and Post-digital Mammographic imaging.

**Figure 1.**
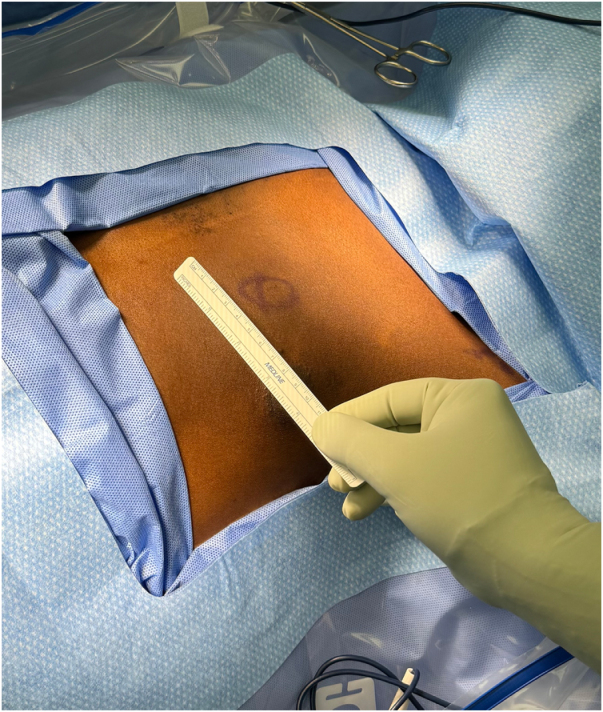
Pre-operative view of lesion location within the left breast. Surgical marker indicative of location, approximately 3 cm medial to the left nipple at the 9-o’clock position.

On examination in the outpatient surgical clinic, a 5 × 4 cm soft, mobile lump was noted in the left breast about 3 cm from the nipple in the 9-o’clock position (see Fig. 1). It did not appear to be adhered to the chest wall or overlying skin. The patient reported minimal discomfort during deep palpation. No visible changes were noted to the overlying skin, nipple, or additional areas of the left breast. No significant findings were appreciated in the right breast, and there was no bilateral axillary lymphadenopathy. Generalized examination did not reveal any features suggestive of neurofibromatosis. Dermatologic examination demonstrated no suspicious findings nor hallmark signs of Café au lait spots, abnormal macules, or other lesions that could be classified as cutaneous neurofibromas. A brief ophthalmic exam did not demonstrate hallmark Lisch nodules nor additional acute or chronic changes. Additionally, no focal neurologic signs were demonstrated during examination.

Per the patient, there was no history of neurofibromatosis in the patient or the known family. Discussions regarding genetic testing were reviewed with the patient, but they declined, given the absence of a concerning history and the presence of a solitary lesion.

Ultrasound-guided biopsy using real-time ultrasound was performed for the concerning circumscribed, spiculated oval mass located in the left breast. A 14-gauge biopsy needle was placed adjacent to the abnormality, and once the needle was documented to be in the correct location, six specimens were obtained using an automated biopsy gun, and a Barrel clip was inserted into the biopsy cavity. Pathology results were consistent with a neurofibroma and were concordant with imaging characteristics of the lesion.

The patient opted for elective surgical excision secondary to discomfort. They were operated on at a nonprofit community hospital in Chicago, Illinois, within the United States of America. The left breast lesion was identified pre-operatively via marking pen and excised under general anesthesia with preservation of the overlying skin. Intraoperatively, the lesion was found to be surrounded by adipose tissue, and the previously placed Barrel clip was identified prior to removal. Macroscopically, a 5 × 4.2 cm lesion was removed that was located about 3 cm from the nipple in the 9-o’clock position down to the pectoral fascia. The semi-rigid lesion of interest was palpable within the adipose covering with an approximate size of 1.6 × 1.4 x 1.2 cm. No direct attachment of the suspected neurofibroma to the overlying dermis or underlying pectoral fascia was noted; connections in the breast appeared to be limited to the adipose envelope. The specimen was sent for confirmatory surgical pathological analysis.

**Figure 2.**
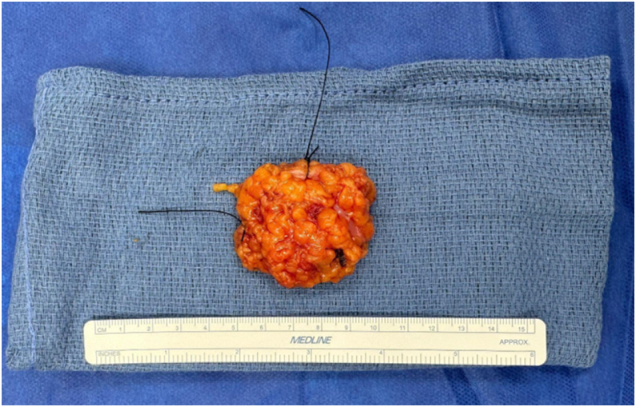
Macroscopic view of resected left breast lesion containing neurofibroma. Suture markings as follows: short side superior, long side lateral. Measured as 5.0 cm (superior to inferior) x 4.2 cm (medial to lateral) x 1.9 cm (anterior to posterior).

Surgical pathology report confirmed diagnosis of a neurofibroma, which was completely excised. The report described a gross excised lesion as 5.0 cm (superior to inferior) x 4.2 cm (medial to lateral) x 1.9 cm (anterior to posterior) with a well-circumscribed tan-white, ovoid lesion of interest internally measuring 1.6 × 1.4 x 1.2 cm (see Fig. 2). Microscopic examination was performed, and findings were incorporated into considerations of the final diagnosis.

**Figure 3.**
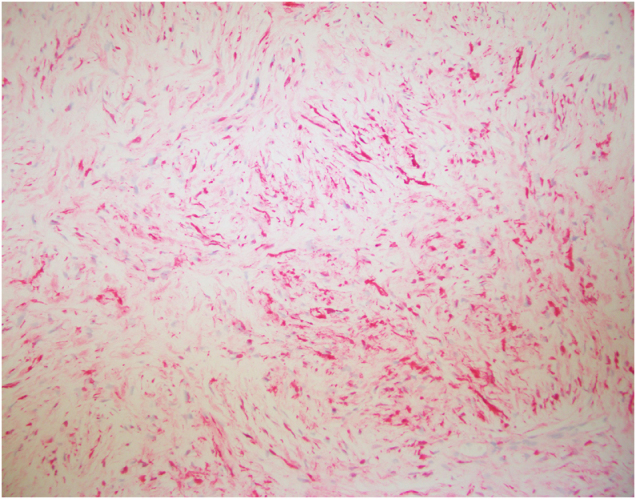
S-100 immunohistochemical stain with a patchy staining pattern.

**Figure 4.**
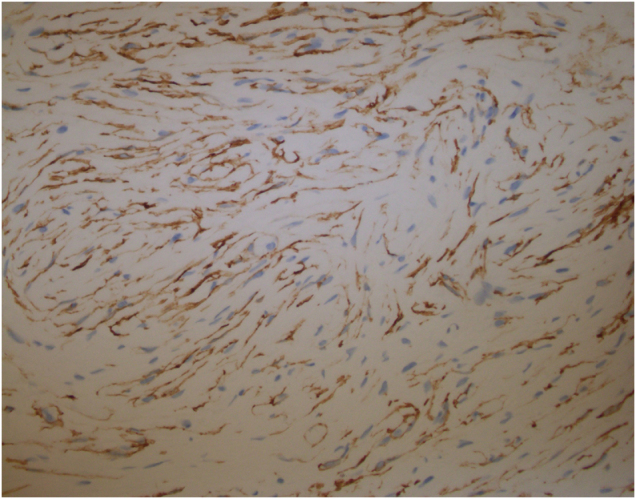
CD34 immunohistochemical stain with whorled bundles in a typical fingerprint-style pattern.

**Figure 5.**
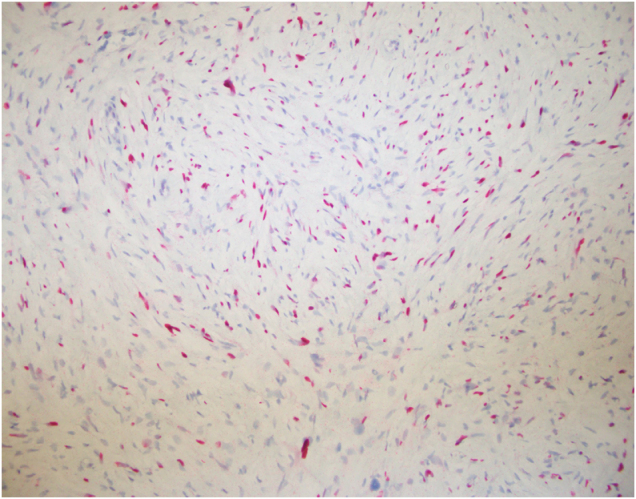
SOX10 immunohistochemical stain positive, a reliable marker of neural crest cell differentiation..

Immunohistochemistry was performed, and figures are provided for positive S-100 (Fig. 3), CD34 markers (Fig. 4), and SOX10 (Fig. 5), all of which are characteristic of neurofibroma. Microscopic images contain characteristic spindle cells with elongated nuclei, and no mitotic activity was appreciated. Additional stains obtained include AE1AE3, CK5-6, Desmin, HMB45, ER, GATA3, Cytokeratin HMW [34βE12], p63, SMA, and Vimentin.

The patient was discharged the same day and seen for follow-up evaluation 1 week post-op in the clinic. At that time, he reported the development of a small fluid pocket, or seroma, which was drained locally. In total, 3 cc of clear fluid was aspirated without notable complications. He denied any localized pain, drainage, excessive bleeding, or other additional symptoms upon the first post-op visit outside of the seroma. Further follow-up appointments were completed at 2 weeks, 4 weeks, and 3 months post-op, with no recurrence of seroma or development of additional complications. Future evaluations were discussed on an as-needed basis until a final visit 1 year post-op. The patient was asked to provide commentary or perspective on surgical intervention and follow-up evaluations, but declined at multiple in-office visits. Please see Table 1 for a list of chronological events.

**Table 1:** Chronological clinical timeline of events

Date (dd/mm/yyyy)	Event	Details
~2019–2021	Initial symptom onset	The patient first noticed a soft, mobile left breast lump while lifting at work. Lump remains stable in size over ~4 years with mild tenderness only on forceful palpation. No nipple or skin changes noted.
15/01/2025	Diagnostic imaging	Bilateral diagnostic digital mammogram and targeted left breast ultrasound performed. Imaging shows a suspicious circumscribed spiculated oval mass in the left breast.
22/01/2025	Ultrasound-guided biopsy	Real-time ultrasound-guided core needle biopsy performed. Six samples were obtained with a 14-gauge biopsy needle; a Barrel clip marker was placed in the biopsy cavity.
Late January 2025	Biopsy pathology finalized	Core biopsy pathology demonstrates neurofibroma, with features concordant with imaging.
13/02/2025	Post-biopsy surgical consultation	Biopsy pathology was reviewed with the patient (neurofibroma). Physical examination reveals a 5 × 4 cm soft, mobile mass in the left breast at the 9-o’clock position. No clinical signs of neurofibromatosis. Genetic testing was discussed and declined. Elective excision planned.
15/04/2025	Surgical excision	Elective excision of the left breast mass was performed under general anesthesia. Gross specimen measures 5.0 × 4.2 × 1.9 cm with an internal well-circumscribed tan-white nodule measuring 1.6 × 1.4 × 1.2 cm. Lesion free from dermal and pectoral fascia attachment. Barrel clip identified intraoperatively.
Late April 2025	Surgical pathology	Surgical pathology confirms a completely excised neurofibroma. IHC positive for S-100, SOX10, and CD34. No mitotic activity observed.
~22/04/2025	1-week postoperative visit	Small seroma noted and aspirated (3 cc clear fluid). No additional complications.
~29/04/2025	2-week postoperative follow-up	Healing appropriate with no seroma recurrence.
~13/05/2025	4-week postoperative follow-up	Continued normal recovery; no complications or recurrence.
~15/07/2025	3-month postoperative visit	Patient asymptomatic. No evidence of recurrence.

## Discussion

Primary tumors of the chest wall are relatively uncommon, with an incidence of less than 2% of the population^[^6,7^]^ and a representation of less than 5% of all thoracic neoplasms^[^8^]^. Within this small fraction, around 50%–85% of these tumors are malignant. Classification of primary tumors by proposed tissue of origin indicates that only 45% arise from soft tissues^[^9^]^.

Within an extensive series of chest wall tumor cases, Po Kuei Hse *et al*^[^8^]^ reported 62 cases of primary tumors of the anterior chest wall without a single neurogenic tumor being described. An additional case series of 20 patients from Malaysia reported only one neurofibroma in a female-majority cohort^[^10^]^. In the largest reported series of neurogenic tumors of the chest wall, the majority of the tumors were located in the posterior chest wall, and only two cases were neurofibromas, with one presenting in a patient with neurofibromatosis type 1 (NF1)^[^11^]^.

Neurofibromas are defined as benign peripheral nerve sheath tumors that originate from the endoneurium, the connective tissue of nerve sheaths^[^12^]^. They are composed of Schwann cells, fibroblasts, perineural cells, and mast cells in a variably myxoid background^[^12^]^. These lesions were first described in 1849 by Smith and von Ricklinghausen in 1882, with the first mention in the breast by John Sherman in 1981^[^3,13,14^]^. Macroscopically, they are well-defined, usually unencapsulated gray, firm masses with gelatinous cut surfaces and attached transected nerve fibers^[^15^]^.

Lesions of this nature are typically associated with the autosomal dominant disorder of neurofibromatosis type 1 (NF1, aka von Recklinghausen disease); however, around 90% of these lesions are sporadic but tend to have NF1 mutations^[^15,16^]^. Most neurofibromas are reported to occur in the head, neck, and extremities, with only a few case reports of mammary neurofibromas in the absence of NF1^[^13,17^]^. Within NF1 itself, the occurrence is still rare, with breast lesions only accounting for 3.5% of all tumors^[^18^]^.

Sporadic neurofibromas have been described as solitary, slow-growing, and typically painless lesions in superficial tissues that typically occur in the third or fourth decade of life without gender bias^[^16,19^]^. With breast location, there is a predilection for the nipple–areolar complex (NAC) or pectoralis fascia^[^16^]^. Three subtypes have been described: localized, diffuse, and plexiform. Of the three, only the diffuse and localized types can occur in the breast^[^16,17,20^]^. The plexiform type typically occurs along deep nerve roots and is pathognomonic of NF1, with only one case reported in the breast^[^18^]^.

Upon extensive literature review and in the context of the information presented above, a solitary neurofibroma of the male breast in the absence of a diagnosis of neurofibromatosis is a substantially unique occurrence^[^1–3^]^. Table 2 summarizes the previously reported cases compared with the case presented here. In this case, the lesion developed as a singular homogenous mass without involvement of the NAC and was devoid of notable deformity of the breast architecture, overlying skin, or the NAC. Current literature suggests surgical excision as the mainstay of management, regardless of their low risk of malignant transformation^[^15^]^. As with other breast lesions, symptomatic or suspicious lesions should undergo surgical excision^[^16^]^. The lack of consistent reported natural history of these lesions across all patient subtypes, in addition to the rarity associated with solitary state in the breast and a male patient, makes it difficult for providers to opt for observation as a management strategy.

**Table 2:** Known reported cases of solitary neurofibromas of the male breast without NF1 diagnosis^[^1–3^]^

Case	Year	Patient presentation	Gross size of excised lesion	Diagnostics	Management	Follow-up
Hock	1995	61-year-old man, left breast, smooth lesion, gradually enlarging over 6 months	Not provided	Fine needle aspiration cytology, Histological examination, H&E, May-Grunwald-Giemsa (MGG).	Lumpectomy	Not provided
Jeyaretna	2007	48-year-old man, left breast, firm lesion, gradually enlarging over 20 years	4 x 3 × 2.5 cm	Fine needle aspiration cytology, core needle biopsy, mammogram image, histological examination, S100: (+)	Excised under general anesthetic	Until 5 years post-op without noted recurrence
Korfias	2013	41-year-old man, right breast, firm lesion, gradually enlarged over a year	12 x 9 × 7 cm	Fine needle aspiration biopsy, core biopsy, histological examination, Vimentin: (+), S100: (+), Desmin: (−), Aktin: (−)	Wide surgical excision	Until 2 years post-op, without noted recurrence
Parkhurst	2025	41-year-old man, left breast, firm lesion, gradually enlarging over 4 years	5.0 x 4.2 × 1.9 cm	Digital diagnostic mammogram, ultrasound-guided needle biopsy, histological examination, S-100: (+), SOX10: (+), CD34: (+)	Lumpectomy	Visits at 1 week, 2 weeks, 4 weeks, and 3 months post-op secondary to seroma development. One-year follow-up planned.

Prognosis is excellent, with very few recurrences reported across lesion sites in patients without NF1; proper margins are the most effective strategy for reducing recurrence rates^[^21^]^. In the three other cases of solitary neurofibroma of the male breast, no recurrence was reported. The rarity of the location has also led to a recommendation of genetic counseling for all mammary neurofibroma patients, including intensive breast cancer surveillance for any concurrently or newly diagnosed NF1 patients^[^16^]^. At this time, no alternative therapies exist for cutaneous neurofibromas^[^22^]^.

Differentials that were considered but ultimately excluded were schwannoma, fibroadenoma, lipoma, breast cyst, and breast cancer. Schwannoma was excluded because there was no established NF2 diagnosis, no distinctive clinical features, and no distinctive Antoni A and Antoni B areas on the reported immunohistological findings. Fibroadenoma was excluded based on the absence of characteristic glandular and stromal tissue on histopathological examination. Lipoma was excluded because there was no evidence of mature adipocytes on histopathology. Breast cyst was ruled out through ultrasound-guided needle biopsy secondary to lack of fluid. Breast cancer was ruled out via diagnostic mammogram, biopsy, and immunohistochemistry.

## Conclusion

Solitary neurofibroma of the male breast in the absence of Neurofibromatosis is an infrequent entity. To our knowledge, this is only the fourth case of its kind and the only one to provide gross imagery and two separate confirmatory pathology reports. Surgical excision remains the recommended management approach, even with relatively low rates of recurrence in general patient populations and no prior recurrence reported in limited cases of solitary lesions within the male breast. Given the rarity of this entity, the approach used for other types of mammary neurofibromas should be considered in all cases, including postoperative genetic counseling and appropriate surveillance. Although significantly uncommon, lesions of similar characteristics should be considered in all male patients with an abnormal breast lesion before confirmatory studies.

## Data Availability

All data is available from the corresponding author upon reasonable request. This case has not been presented or shared at any conference or regional meeting.

## References

[1] Hock YL Mohamid W. Myxoid neurofibroma of the male breast: fine needle aspiration cytodiagnosis. Cytopathology 1995;6:44–47. 7734701 10.1111/j.1365-2303.1995.tb00007.x

[2] Jeyaretna DS Oriolowo A Smith ME. Solitary neurofibroma in the male breast. World J Surg Oncol 2007;5. doi:10.1186/1477-7819-5-23 PMC1808458 17324294

[3] Korfias D Tsikkinis A Kondi-Paphitis A. Rare case of a large tumour of the breast area in 41-year-old male. Hell J Surg 2013;85:113–15.

[4] Tevatia MS Mishra P Baranwal AK. Primary Breast Tumors with Mesenchymal Morphology. J Lab Physicians 2021;13:362–67. 34975257 10.1055/s-0041-1732492 PMC8714411

[5] Kerwan A Al-Jabir A Mathew G. Revised Surgical CAse REport (SCARE) guideline: An update for the age of Artificial Intelligence. Premier J Sci 2025;10:100079.

[6] Faber LP. Chest Wall Tumors: Introduction. Semin Thorac Cardiovasc Surg 1999;11:250. 10451256 10.1016/s1043-0679(99)80005-7

[7] Incarbone M Pastorino U. Surgical Treatment of Chest Wall Tumors. World J Surg 2001;25:218–30. 11338025 10.1007/s002680020022

[8] Hsu PK Hsu HS Lee HC. Management of Primary Chest Wall Tumors: 14 Years’ Clinical Experience. J Chin Med Assoc 2006;69:377–82. 16970274 10.1016/s1726-4901(09)70276-x

[9] Cipriano A Burfeind W. Management of Primary Soft Tissue Tumors of the Chest Wall. Thorac Surg Clin 2017;27:139–47. 28363368 10.1016/j.thorsurg.2017.01.007

[10] Dharmaraj B Diong NC Shamugam N. Chest wall resection and reconstruction: a case series of 20 patients in Hospital Kuala Lumpur, Malaysia. Indian J Thorac Cardiovasc Surg 2020;37:82–88. 10.1007/s12055-020-00972-7 PMC7778637 33442211

[11] Yamaguchi M Yoshino I Fukuyama S. Surgical treatment of neurogenic tumors of the chest. Ann Thorac Cardiovasc Surg 2004;10:148–51. 15312009

[12] Ferner RE OʼDoherty MJ. Neurofibroma and schwannoma. Curr Opin Neurol 2002;15:679–84. 12447105 10.1097/01.wco.0000044763.39452.aa

[13] Thompson S Kaplan SS Poppiti RJ. Solitary neurofibroma of the breast. Radiol Case Rep 2012;7:462. 27330588 10.2484/rcr.v7i4.462 PMC4899552

[14] Sherman JE Smith JW. Neurofibromas of the Breast and Nipple-Areolar Area. Ann Plast Surg 1981;7:302–07. 6797340 10.1097/00000637-198110000-00010

[15] Messersmith L Krauland K. Neurofibroma. PubMed. Published 2020. Accessed April 30, 2025. https://www.ncbi.nlm.nih.gov/books/NBK539707/

[16] Yin C Porembka JH Hwang H. Neurofibroma in the Breast: Diagnosis and Management Considerations. J Breast Imaging 2021;3:363–68. 38424772 10.1093/jbi/wbab008

[17] Rotili A De Maria F Di Venosa B. Solitary breast neurofibroma: imaging aspects. Ecancermedicalscience 2018;12. doi:10.3332/ecancer.2018.800 PMC5813918 29456617

[18] Bongiorno MR Doukaki S Aricò M. Neurofibromatosis of the nipple-areolar area: a case series. J Med Case Rep 2010;4. doi:10.1186/1752-1947-4-22 PMC2823760 20205809

[19] Russell DH Montgomery EA Susnik B. Low to Intermediate (Borderline) Grade Breast Spindle Cell Lesions on Needle Biopsy: Diagnostic Approach and Clinical Management. Adv Anat Pathol 2022;29:309–23. 35838633 10.1097/PAP.0000000000000353

[20] Nasri S Benmoussa Y Abbou W. Imaging appearance of isolated diffuse neurofibroma of nipple areolar area: a case report. Pan Afr Med J 2021;39. Accessed April 30, 2025. https://www.ajol.info/index.php/pamj/article/view/221437 10.11604/pamj.2021.39.178.30438 PMC8449569 34584604

[21] Azam R Mrkonjic M Gupta A. Mesenchymal Tumors of the Breast: Fibroblastic/Myofibroblastic Lesions and Other Lesions. Curr Oncol 2023;30:4437–82. 37232796 10.3390/curroncol30050338 PMC10217748

[22] Allaway RJ Gosline SJC La Rosa S. Cutaneous neurofibromas in the genomics era: current understanding and open questions. Br J Cancer 2018;118:1539–48. 29695767 10.1038/s41416-018-0073-2 PMC6008439

